# Prehospital identification of sepsis patients and alerting of receiving hospitals: impact on early goal-directed therapy

**DOI:** 10.1186/cc10395

**Published:** 2011-10-27

**Authors:** K Halimi, J Freeman-Garrick, C Agcaoili, K Choy, F Claridge, M Jacobs, M Taigman

**Affiliations:** 1 Department of Emergency Medicine and Intensive Care, Washington Hospital, Fremont, CA, USA; 2 Highland Hospital, Oakland, CA, USA; 3 Alameda County Emergency Medical Services, San Leandro, CA, USA; 4 American Medical Response, Alameda County, CA, USA

## Introduction

Over the past several years, the early identification and aggressive treatment of sepsis patients has become a standard of care in the hospital setting. A relatively small number of emergency medical service (EMS) systems have started programs to screen for sepsis; an even smaller number provide treatment based on that screening process in the prehospital setting.

## Objective

The purpose of this study is twofold. First, the study aims to determine how effectively paramedics working in the county EMS system can use a screening tool to identify potential sepsis patients and provide an alert to the receiving hospital. Second, the study will examine whether or not an early identification process in the field leads to improved treatment of sepsis. The end goal is to reduce morbidity and mortality of sepsis patients in the hospital setting.

## Methods

This is a multi-site prospective observational study with comparison to retrospective cohort. Patient data will be collected to determine whether or not the alert process leads to early obtaining of a serum lactate measurement and early goal-directed therapy.

## Results

Data points being analyzed from prehospital care reports: criteria from the sepsis screening tool include evidence of infection, temperature, heart rate, respiratory rate; and EMS field clinical impression (for comparison with emergency department (ED) admitting diagnosis). Data points being analyzed in the hospitals include the following: ED admitting diagnosis; serum lactate values, blood culture, timestamps; evidence of early goal-directed therapy - timestamps/values for fluid and antibiotic administration; hospital admitting diagnosis; and discharge diagnosis. The results of the study will be available by 2012/2013.

## Conclusion

Preliminary anecdotal and early data analysis reports from EMS and hospital staff suggest that patients are being identified as septic prior to ED arrival and have lower lactate levels. Patients are also treated more timely on arrival to the ED for sepsis. Final data analysis will shed more light on our hypothesis. Early identification of septic patients has implications for further research both in the field and hospital settings.

